# Synergistic Effect of Polysaccharides from Chamomile Tea with Nitazoxanide Increases Treatment Efficacy against *Giardia intestinalis*

**DOI:** 10.3390/life12122091

**Published:** 2022-12-13

**Authors:** Bruna Sabatke, Pedro Felipe P. Chaves, Lucimara M. C. Cordeiro, Marcel I. Ramirez

**Affiliations:** 1 Graduate Program in Microbiology, Parasitology and Pathology, Federal University of Paraná, Curitiba 81531-980, PR, Brazil; 2 EVAHPI—Extracellular Vesicles and Host-Parasite Interactions Research Group, Laboratório de Biologia Molecular e Sistemática de Tripanossomatideos, Carlos Chagas Institute-Fiocruz, Curitiba 81310-020, PR, Brazil; 3 Department of Biochemistry and Molecular Biology, Federal University of Paraná, Curitiba 81531-980, PR, Brazil

**Keywords:** chamomile tea, polysaccharides, *Giardia intestinalis*, antiparasitic drugs, alternative treatment, synergism

## Abstract

*Giardia intestinalis* (syn. *G. lamblia, G. duodenalis*) is a protozoa parasite that produces one of the most frequent waterborne causes of diarrhea worldwide. This protozoan infects most mammals, including humans, and colonizes the small intestine, adhering to intestinal cells. The mechanism by which *G. intestinalis* causes diarrhea is multifactorial, causing intestinal malabsorption. The treatment of giardiasis uses chemotherapeutic drugs such as nitroimidazoles, furazolidone, paromomycin, and benzimidazole compounds. However, they are toxic, refractory, and may generate resistance. To increase efficacy, a current treatment strategy is to combine these drugs with other compounds, such as polysaccharides. Several studies have shown that polysaccharides have gastroprotective effects. Polysaccharides are high-molecular weight polymers, and they differ in structure and functions, being widely extracted from vegetables and fruits. In the present study, we show that polysaccharides found in chamomile tea (called MRW), in contact with antiparasitic agents, potentially inhibit the adhesion of parasites to intestinal cells. Moreover, at 500 µg/mL, they act synergistically with nitazoxanide (NTZ), increasing its effectiveness and decreasing the drug dose needed for giardiasis treatment.

## 1. Introduction

*Giardia intestinalis* (syn. *G. lamblia*, *G. duodenalis*) is a protozoa parasite that produces one of the most frequent waterborne causes of diarrhea worldwide [1]. *G. intestinalis* produces self-limiting diarrhea, occurring mainly in endemic places with basic sanitation problems, which increases the chances of reinfection. The treatment for giardiasis is conducted with several drugs, with differences in efficacy and, in some cases, with resistance, which has been reported in chronic patients [2]. The most used compounds are derivatives of 5-nitroimidazoles (5-NIs) and benzimidazoles (BIs), quinacrine, furazolidone, paromomycin, and nitazoxanide [3]. These agents have distinct modes of action, and involve a variety of labeling and processing mechanisms that affect trophozoite cellular processes [2]. Paromomycin and quinacrine are eventually used to treat giardiasis, but they have limitations due to low efficacy and high toxicity. The effectiveness of treatment with metronidazole ranges from 73 to 100%, and from 79 to 100% for albendazole [4,5]. However, these agents are often associated with adverse side effects and therapeutic failures, more frequently due to poor adherence to treatment chemotherapy, immunosuppression, immune deficiency, reinfection, or drug resistance to metronidazole-related compounds [3,6,7]. Therefore, there is a growing interest in finding new strategies and compounds against *Giardia* that are non-toxic and do not cause parasite resistance [8].

Nitazoxanide (NTZ) is a derivative of a nitrothiazolyl-salicylamide broad-spectrum antiparasitic agent known to be effective in the treatment of protozoal and helminthic intestinal infections. In vitro studies have shown that NTZ inhibits pyruvate ferredoxine oxidoreductase (PFOR), an essential enzyme in the anaerobic metabolism of the parasite [9,10,11]. Cedillo-Rivera et al (2002) [12] demonstrated the greater efficacy of NTZ than metronidazole against *G. intestinalis*. In addition to being more effective, the treatment non-toxic and produces fewer side effects; thus, NTZ is an alternative drug for the treatment of infectious diarrhea [13]. 

An alternative strategy to increase the treatment efficacy is the search for natural products, polymers, or macromolecules, such as polysaccharides, that could have synergistic effects on the treatment of parasitosis [14].

Polysaccharides are high-molecular weight polymers, consisting of at least ten monosaccharides joined by glycosidic linkages. Polysaccharides isolated from natural resources have shown pharmacological activities, such as anti-inflammatory, antitumor, immunomodulator, antioxidant, sedative, and anxiolytic-like effects [15,16,17,18,19]. 

Some authors have already demonstrated the synergistic effect of polysaccharides with drugs as a new alternative strategy against other infectious agents. Lo et al. [20], demonstrated that chitosan, in combination with fluconazole, showed a great synergistic fungicidal effect against *C. albicans* and *C. tropicalis.* In the study published by Ganan et al. [21], it was shown that oligomers of chitosan, known as chito-oligosaccharides, combined with commercial antifungals, were capable of inhibiting yeast strains.

Previously, we have chemically characterized polysaccharides from *Chamomilla* or *Matricaria recutita* [L.] Rauschert tea, commonly known as German chamomile [22]. It is consumed as an infusion (tea) due to its gastrointestinal effects, and is also considered an anti-inflammatory medicine [23,24]. The polysaccharides found in chamomile tea (called MRW) are composed mainly of a mixture of inulin-type fructan and highly methyl esterified and acetylated homogalacturonan (degree of methylesterification of 87% and degree of acetylation of 19%), with small amounts of type II arabinogalactan and an acid xylan [22].

In the present study, we show that chamomile polysaccharides act synergistically with nitazoxanide (NTZ), increasing its efficacy, and suggesting an alternative treatment of the giardiasis.

## 2. Materials and Methods

### 2.1. Chamomile Tea Polysaccharides

Chamomile tea polysaccharides were extracted and chemically characterized by Chaves et al. [22]. Briefly, the chamomile tea was prepared from the floral chapters by infusion. The tea was filtered and concentrated under reduced pressure, and the polysaccharides were precipitated with 95% ethanol (3 vol.). The polysaccharides were recovered by filtration, dialyzed in a semipermeable membrane (Cellulose Spectrumlabs 6–8 kDa cut-off), and freeze-dried, giving the MRW fraction (*Matricaria recutita* water extract) that was used in the experiments in the present study.

### 2.2. G. intestinalis Isolates and Caco-2 Cell Culture

Isolate WB (ATCC 50803) was grown in TYI S-33 medium [25], and supplemented with 10% heat inactivated adult bovine serum (ABS) with 1% penicillin/streptomycin 1000U (Gibco^TM^, Waltham, MA, USA) and 0.5 mg/mL bovine bile (Thermo Fisher^TM^, Waltham, MA, USA) at 37 °C under microaerophilic conditions. The cultures were maintained in polystyrene tubes (BD Biosciences^TM^, San Jose, CA, USA) (13 mL until confluence (1 × 10^6^ cells/mL)) and subcultured thereafter, each for 72 h. Human colorectal adenocarcinoma cells, Caco-2 cells (ATCC CRL-2102), were cultured in RPMI-1640 supplemented with 10% fetal bovine serum (FBS) and 1% penicillin/streptomycin 1000U (Gibco^TM^). Cells were incubated at 37 °C in 5% CO_2_ until a confluent cell monolayer was reached. Caco-2 Cells were utilized in experiments to analyze the adherence of *Giardia* to the cells in the presence of anti-protozoan drugs and fraction MRW. 

### 2.3. Trophozoites Growth Curves

We evaluated whether trophozoite growth was altered in the presence of the MRW fraction. A 5 × 10^4^ total inoculum was resuspended in TYI-S-33 medium (polystyrene culture tubes), in the absence (CTL) or presence of MRW fraction extracted from chamomile tea (250 or 500 µg/mL), and incubated at 37 °C for 72 h. We began the growth curve with 1.25 × 10^4^/mL, and every 24 h, the tubes were cooled and aliquots of 10 µL were removed from the culture tubes to count the trophozoites in a hemocytometer under an optical microscope. 

### 2.4. Cell Viability 

Caco-2 cells were seeded in 24-well plates at a density of 1 × 10^5^ cells per well, and incubated for 24 h. Then, the cells were exposed to 250 or 500 µg/mL of MRW fraction for 24 h. After the exposure time, the cells were washed 2 times with RPMI. The viability index was calculated as the ratio of living cells to the total number of cells. Dead cells were detected by trypan blue dye staining counted in a microscope. The analyses were performed in triplicate. The percentage of viable (unstained) and non-viable (stained in blue) cells was calculated according to the following equation:

Viable cells = (Total viable cells/Total cells counted) × 100

### 2.5. In Vitro Cytotoxic Effect

The active compound (NTZ) was evaluated for its toxicity against mammalian Caco-2 cells, showing an CC50 of 227.5 µM. The selectivity index (SI) of the compound, defined as the ratio of cyto-toxicity to biological activity (SI = CC50 VERO cells/IC50 parasites) was calculated. It is generally considered that biological efficacy is not due to in vitro cytotoxicity when SI ≥ 10 [26].

### 2.6. Adhesion Assay of Parasite in the Presence of MRW Fraction

We seeded 1 × 10^5^ Caco-2 intestinal cells in 24 well plate at 37 °C, and they were incubated until reaching confluence. Then, cells were incubated in the absence (CTL) or presence of MRW fraction extracted from chamomile tea, at 125, 250, or 500 µg/mL for 1 h at 37 °C. Then, 5 × 10^5^ trophozoites of *Giardia intestinalis* were added and incubated for 1 h at 37 °C to assess whether the polysaccharides could alter the ability of parasites to bind to the cells following the procedure adapted from Evans-Osses et al. [27]. After the incubation period (1 h), the adhesion was estimated by counting non-adherent parasites in a Neubauer chamber in the supernatant, and this number was subtracted from the total number of inoculated parasites.

### 2.7. Synergism between Antiprotozoal Drugs and MRW Fraction

The trophozoites 5 × 10^5^ (cultured under axenic conditions) were added into 5 mL tubes containing TYI-S-33 medium, supplemented with ABS and an antibiotic (penicillin/streptomycin 1000U). Trophozoites were maintained in the presence of 250 or 500 µg/mL of MRW and/or NTZ + polysaccharide, following the procedure adapted from Velázquez-Olvera et al. [14]. The chamomile and NTZ structures were defined previously by [22,28]). Cultures were incubated for 48 h at 37 °C, and every 24 h, 10 µL was taken to count the trophozoites. All assays were performed in triplicate, with the medium, *Giardia,* and nitazoxanide as controls.

### 2.8. G. intestinalis Trophozoite Recovery Assay

*G. intestinalis* trophozoites (approximately 1–8 × 10^5^) from the assay described above (after 48 h) were resuspended in new TYI-S-33 culture medium, then supplemented with ABS and antibiotic in 5 mL tubes following the procedure adapted from Abraham et al. [29]. A growth curve was restarted by incubating the tubes at 37 °C. The assay was followed for 72 h and quantified every 24 h using a hemocytometer. 

### 2.9. Statistical Analysis

Statistical analysis of the data was performed with GraphPad Prism 6 Software using the one or two-way ANOVA test. Values are represented as means ± standard errors of the means (SEM), acquired in biological triplicates. The normality of the data was assessed prior to analysis. *p* < 0.05 was defined as statistically significant.

## 3. Results 

**Figure 1:**
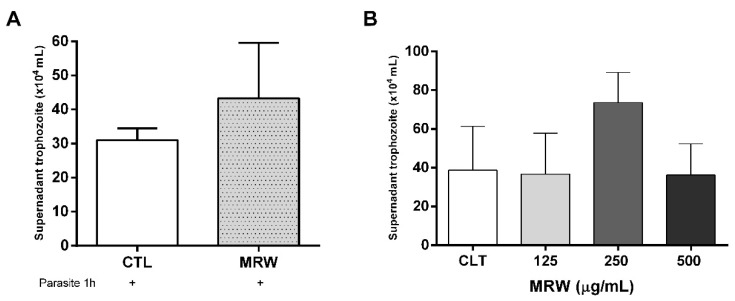
Adhesion assay of parasites in the presence of MRW fraction. (**A**) Number of trophozoites present in supernatant of Caco-2 cells after incubation with 250 μg/mL MRW fraction after 1 h. (**B**) Quantification of trophozoites in Caco-2 cell supernatant after incubation with MRW fraction. Total non-adherent parasites in Caco-2 cells infected with *G. intestinalis*, in the absence (CTL) and post-treatment of 125, 250, and 500 μg/mL MRW. There is no difference in the comparison of statistical significance.

Natural products extracted from plants represent a therapeutic alternative, often used to reduce the toxicity of drugs and increase their efficacy. Conventional treatments of giardiasis have proved to be toxic, producing heterogeneous responses and parasite resistance. Our first assay was to incubate Caco-2 intestinal cells with 250 µg/mL MRW fraction extracted from chamomile tea, and then to add trophozoite forms of *G. intestinalis* in order to assess whether the polysaccharides could alter the ability of parasites to bind to the cells. Figure 1A shows a preliminary assay, where chamomile tea polysaccharides (fraction MRW) inhibited the adhesion of *Giardia* to intestinal cells. Further, we investigated whether the polysaccharides could have a dose-dependent effect of MRW fraction on the ability of *Giardia* to adhere to Caco-2 cells. As can be seen in Figure 1B, only the concentration of 250 µg/mL inhibited the trophozoite adhesion to intestinal cells, as shown in Figure 1A. In both results, there was no significant difference between the control and the presence of the MRW fraction. Moreover, during that time, the cells’ viability was unchanged.

**Figure 2:**
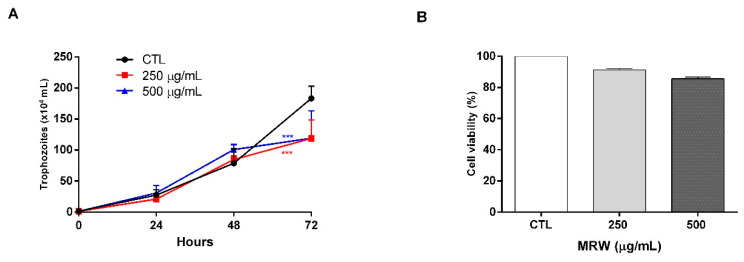
Effects of polysaccharides on parasites and cells. (**A**) Growth curve of the *G. intestinalis* culture after 72 h of incubation with 250 or 500 µg/mL MRW fraction. (**B**) Viability of Caco-2 cells after incubation with the MRW fraction for 72 h. *** Statistically different from control (*p* < 0.009).

Subsequently, we decided to evaluate whether the chamomile polysaccharides could be altering the proliferation of the parasites. For this reason, we began a growth curve of the parasites in the presence of different concentrations of MRW fraction. For up to 48 h, no effect was seen at either tested concentration (250 and 500 µg/mL). However, at 72 h, we observed a lower growth rate of the parasites incubated with polysaccharides in relation to the control (Figure 2A). Then, the effect of MRW on the viability of intestinal Caco-2 cells was investigated (Figure 2B). Results showed that none of the MRW concentrations significantly altered the viability of these cells (Figure 2B). 

**Figure 3:**
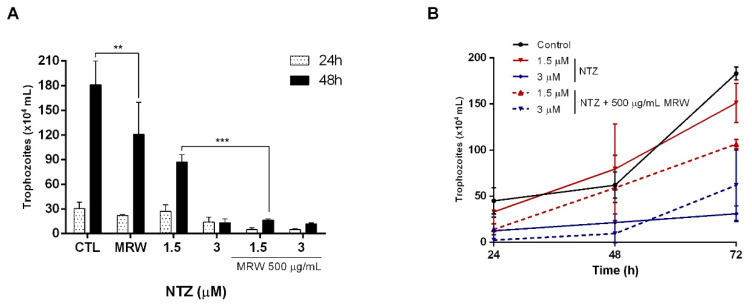
In vitro synergistic effects between NTZ and MRW fraction against *G. intestinalis.* (**A**) In vitro synergistic effects between NTZ and 500 µg/mL MRW fraction in 48 h. (**B**) Reestablishment of the parasite growth curve after synergism assay with NTZ and 500 µg/mL MRW fraction. ** Statistically different from control (*p* < 0.001). *** Statistical significance from 1.5 µM NTZ and MRW (*p* < 0.001).

An alternative to increase the efficacy of anti-*Giardia* drugs is synergism. We performed tests with NTZ to evaluate its inhibition effect against *G. intestinalis,* either in vitro alone or in combination with MRW. The results showed an inhibition of parasite growth when treated with 1.5 μM of NTZ in the presence of 500 μg/mL of MRW fraction, and an increase of at least five times in the treatment effectiveness when compared with NTZ alone, indicating a synergistic effect between the compounds at 48 h (Figure 3A). These results, as well as the selectivity data of the drug (SI ≥ 250—not shown) reinforced the need to test lower concentrations of NTZ that could demonstrate the synergistic effect of the treatment. However, no major synergistic effects were observed at concentrations below 1.5 μM (not shown). 

Due to the presence of resistance, which can make infections chronic, an important parameter is to evaluate the ability of the parasite to recover after being incubated with the combination of drugs and polysaccharides. Figure 3B shows that parasites incubated with 1.5 µM of NTZ seemed to recover their growth curve, while parasites that were incubated with the same concentration of the drug and the MRW fraction showed a curve lower than the negative control (no drug) and the drug alone. Taken together, these results suggest that NTZ plus MRW should be used as a new strategy for the treatment of giardiasis, since some concentrations of the antiparasitic, when combined with the fraction MRW, are able to reduce the recovery of the parasites’ culture.

## 4. Discussion

Giardiasis is one of the most common parasitic diarrheal diseases worldwide, affecting thousands of people. Several drugs are available for the treatment of this parasitosis, but unfortunately, all of them have variable efficacy and adverse effects, probably due to inadequate doses or poor adherence to treatment, immunosuppression, reinfection, or drug resistance [3]. The toxicity of treatment-refractory drugs and the occurrence of metronidazole-resistant *G. intestinalis* have motivated the search for other drugs as alternatives. The NTZ emerged as an alternative, as did the combination of treatments with drugs and other components to decrease toxicity and increase efficacy [2]. Given the above, therapeutic alternatives have become very attractive, such as the search for natural products, polymers, or macromolecules such as polysaccharides that could have synergistic effects in the treatment of giardiasis.

We conducted our study using polysaccharides extracted from chamomile tea (*Chamomilla recutita*). Chamomile tea belongs to an important group of cultivated medicinal plants, often referred to as the “star among medicinal species”. More than 120 chemical constituents have been identified in chamomile as secondary metabolites, which gives chamomile tea its multi-therapeutic, cosmetic, and nutritional values, which have been established through years of traditional and scientific use and research [30]. The presence of inulin, FOS, highly methyl-esterified homogalacturonan, type II arabinogalactan, and acidic xylan in chamomile tea shows that secondary metabolites may be the molecules responsible for the health benefits of its consumption, and also adds a new property to chamomile as a source of structurally diverse dietary fiber with potential prebiotic, gastrointestinal, and immunological functions.

In our trials, we evaluated the effect of the recently described MRW fraction from chamomile tea in the adhesion of parasites and synergism (combination of MRW with NTZ). We have demonstrated that the presence of MRW inhibited the adhesion of *Giardia* to intestinal cells. Furthermore, the concentration of 1.5 μM NTZ, combined with 500 μg/mL MRW polysaccharide, was able to potentiate anti-giardia activity. It was demonstrated that addition of MRW fraction to 1.5 μM NTZ reduced the growth of parasites by nearly five times when compared to NTZ alone. We have also demonstrated that *Giardia* presented a lower growth curve recovery when compared with parasites that were not incubated with MRW. However, the mechanisms of action of MRW fraction against *G. intestinalis* have not been identified. The MRW fraction is mainly composed of inulin and pectic polysaccharides (homogalacturonan domain), with small amounts of type II arabinogalactan and acid xylan.

The effects of inulin supplementation in a model of malnourished murine giardiasis have already been reported by Shukla et al. [31]. It was observed that inulin supplementation either prior (7 days) or simultaneously (9 days) with *Giardia* infection in malnourished mice significantly reduced the severity of giardiasis and increased the body and small intestine mass, along with increased lactobacilli counts in feces compared with malnourished *Giardia*-infected mice. Inulin modulated the immune response (increased anti-giardial IgA and IgG, IL-6, IL-10, and nitric oxide levels, and decreased TNF-α levels) and restored the gut morphology. Recently, the combination of inulin with probiotics or probiotics + NTZ were also tested as prophylactic (7 days prior to the infection) and therapeutic agents (at 22 days) against giardiasis in a murine model [32]. There was a significant reduction in *Giardia* cyst shedding and a remarkable improvement of histopathological findings in the small intestine, with a reduction in caspase-3 apoptotic activity and an increase in IL-6 levels, especially after the addition of combined treatment (inulin + probiotics + NTZ). In these studies, it seems that inulin exerts its anti-infective activity indirectly, by stimulating the composition and/or activity of the gut microbiota (microbiota-dependent mechanisms), which may cause a broad array of effects that confer higher colonization resistance on the host. However, complex polysaccharides/prebiotics may also have anti-infective activities against bacteria, protozoa, and viruses by interaction with the immune system of the host or with the pathogens themselves (microbiota-independent mechanisms) [33]. In the latter case, mechanisms include the blocking of pathogen adhesion sites in the gut; since prebiotics also have a carbohydrate structure, they could act as soluble receptor analogues, thereby disrupting the microbial lectin–host receptor interactions and dislodging the adherent pathogen from the gut without it infecting the host [34,35]. The anti-adhesive properties of probiotics must be demonstrated, and there are several possible blocking mechanisms which have been reviewed by Shoaf-Sweeney and Hutkins [35].

Sousa et al. [36] verified that the attachment of *Giardia* trophozoites to Int-407 human intestinal cells may be mediated by a combination of a specific recognition of host cells by surface lectins and mechanical/hydrodynamic forces. Thus, the action of polysaccharides present in the MRW fraction by this mechanism cannot be ruled out, and should be further investigated. Jantscher-Krenn et al. [37] observed that human milk oligosaccharides (HMO) and galactooligosaccharides inhibited the attachment and cytotoxicity of the protozoan *Entamoeba histolytica* to enteric cell layers (HT-29 cells) in vitro, in a dose-dependent manner. This study was structure-specific, since a free terminal Gal was necessary for the activity. Thus, the authors speculated that HMO may bind to Gal/GalNAc lectin on the surface of *E. histolytica* trophozoites. Interestingly, the authors commented that HMO did not affect attachment or viability of *Giardia*. It is important to point out that HMO has a different chemical structure when compared with polysaccharides present in the MRW fraction. 

Several other prebiotics with the ability to inhibit the adhesion of different bacteria, viruses, and parasites to epithelial cells (Caco-2, T84, HT-29, etc.) in vitro were summarized by Azagra-Boronat et al. [33]. Pectic oligosaccharides inhibited the adhesion of pathogenic *Escherichia coli* strains to human intestinal HT-29 cells in vitro [38], while an acidic homogalacturonan-rich structure in carrot water-soluble pectin was reported to block adhesion of pathogenic *E. coli* to uroepithelial cells [39]. Receptor mimicry is unlikely to be the mechanism behind the antiadhesive effect, and further investigation is needed to answer this question [40]. Another suggested mechanism by which prebiotics may alter the adhesive potential is by downregulating the expression of adhesion-related genes, as observed in the case of *Listeria monocytogenes* [41,42]. Thus, both inulin and pectic polysaccharides present in the MRW fraction may be responsible for the antiparasitic activity, probably acting via diverse mechanisms which are not yet fully understood.

Finally, our data reinforce the importance of carrying out other trials to explore a possible clinical application that would allow for the use of a lower dose of NTZ combined with polysaccharides, which, consequently, would lead to fewer undesirable side effects. In addition to these, we intend to investigate the role of the MRW fraction and its antiparasitic effect in vivo, as well as to follow the interactions of the compounds in *Giardia* metabolism.

## 5. Conclusions

Our data demonstrated that the combination of MRW fraction with antiparasitic agents such as NTZ potentially inhibits the adhesion of parasites to intestinal cells. This treatment also decreases the reestablishment of new cultures, suggesting the use of polysaccharides as a treatment alternative. Moreover, this study adds another important aspect to chamomile with regard to its therapeutic properties, making the treatment for *G. intestinalis* less toxic and more effective.

## Data Availability

Not applicable.

## References

[1] Adam R.D. (2001). Biology of *Giardia lamblia*. Clin. Microbiol. Rev..

[2] Argüello-García R., Leitsch D., Skinner-Adams T., Ortega-Pierres M.G. (2020). Drug resistance in Giardia: Mechanisms and alternative treatments for Giardiasis. Adv. Parasitol..

[3] Lalle M., Hanevik K. (2018). Infection and Drug Resistance Dovepress Treatment-refractory giardiasis: Challenges and solutions. Infect. Drug Resist..

[4] Gardner T.B., Hill D.R. (2001). Treatment of giardiasis. Clin. Microbiol. Rev..

[5] Solaymani-Mohammadi S., Singer S.M. (2011). Host immunity and pathogen strain contribute to intestinal disaccharidase impairment following gut infection. J. Immunol..

[6] Leitsch D. (2015). Drug Resistance in the Microaerophilic Parasite *Giardia lamblia*. Curr. Trop. Med. Rep..

[7] Upcroft P., Upcroft J.A. (2001). Drug targets and mechanisms of resistance in the anaerobic protozoa. Clin. Microbiol. Rev..

[8] Nash T.E. (2001). Treatment of *Giardia lamblia* infections. Pediatr. Infect. Dis..

[9] Miyamoto Y., Eckmann L. (2015). Drug Development against the Major Diarrhea-Causing Parasites of the Small Intestine, *Cryptosporidium* and *Giardia*. Front. Microbiol..

[10] White A.C. (2004). Nitazoxanide: A new broad spectrum antiparasitic agent. Expert Rev. Anti-Infect. Ther..

[11] Fox L.M., Saravolatz L.D. (2005). Nitazoxanide: A New Thiazolide Antiparasitic Agent. Clin. Infect. Dis..

[12] Cedillo-rivera R., Chavez B., Gonzalez-Robles A., Tapia A., Yepez-Mulia L. (2022). In Vitro Effect of Nitazoxanide against *Entamoeba histolytica*, *Giardia intestinalis* and *Trichomonas vaginalis* Trophozoites. J. Eukaryot. Microbiol..

[13] Hashan M.R., Elhusseiny K.M., Huu-Hoai L., Tieu T.M., Low S.K., Minh L.H.N., Nghia T.L.B., Loc L.Q., Y M.N., Eid P.S. (2020). Effect of nitazoxanide on diarrhea: A systematic review and network meta-analysis of randomized controlled trials. Acta Trop..

[14] Velázquez-Olvera S., Salgado-Zamora H., Jiménez-Cardoso E., Campos-Aldrete M.E., Pérez-González C., Bem Hadda T. (2016). In vitro anti-*Giardia lamblia* activity of 2-aryl-3-hydroxymethyl imidazo [1,2-a] pyridines and -pyrimidines, individually and in combination with albendazole. Acta Trop..

[15] Cho C.W., Han C.J., Rhee Y.K., Lee Y.C., Shin K.S., Shin J.S., Lee K.T., Hong H.D. (2015). Cheonggukjang polysaccharides enhance immune activities and prevent cyclophosphamide-induced immunosuppression. Int. J. Biol. Macromol..

[16] Chaves P.F.P., Hocayen P.A.S., Dallazen J.L., de Paula Werner M.F., Iacomini M., Andreatini R., Cordeiro L.M.C. (2020). Chamomile tea: Source of a glucuronoxylan with antinociceptive, sedative and anxiolytic-like effects. Int. J. Biol. Macromol..

[17] Li L.J., Li M.Y., Li Y.T., Feng J.J., Hao F.Q., Lun Z. (2012). Adjuvant activity of Sargassum pallidum polysaccharides against combined Newcastle disease, infectious bronchitis and avian influenza inactivated vaccines. Mar. Drugs.

[18] Li N., Li L., Fang J.C., Wong J.H., Ng T.B., Jiang Y., Wang C.R., Zhang N.Y., Wen T.Y., Qu L.Y. (2012). Isolation and identification of a novel polysaccharide-peptide complex with antioxidant, anti-proliferative and hypoglycaemic activities from the abalone mushroom. Biosci. Rep..

[19] Tamiello C.S., Adami E.R., de Oliveira N.M.T., Acco A., Iacomini M., Cordeiro L.M.C. (2018). Structural features of polysaccharides from edible jambo (*Syzygium jambos*) fruits and antitumor activity of extracted pectins. Int. J. Biol. Macromol..

[20] Lo W.H., Deng F.S., Chang C.J., Lin C.H. (2020). Synergistic Antifungal Activity of Chitosan with Fluconazole against *Candida albicans*, *Candida tropicalis*, and Fluconazole-Resistant Strains. Molecules.

[21] Ganan M., Lorentzen S.B., Aam B.B., Eijsink V.G.H., Gaustad P., Sørlie M. (2019). Antibiotic saving effect of combination therapy through synergistic interactions between well-characterized chito-oligosaccharides and commercial antifungals against medically relevant yeasts. PLoS ONE.

[22] Chaves P.F.P., Iacomini M., Cordeiro L.M.C. (2019). Chemical characterization of fructooligosaccharides, inulin and structurally diverse polysaccharides from chamomile tea. Carbohydr. Polym..

[23] Lorenzi H., de A Matos F.J. (2008). Plantas Medicinais Do Brasil Nativas E Exóticas.

[24] Sousa M.P., Matos M.E.O., Matos F.J.A., Machado M.I.L., Craveiro A.A. (1991). Constituintes Químicos Ativos de Plantas Medicinais brasileiras.

[25] Keister D.B. (1983). Axenic Culture of *Giardia lamblia* in TYI-S-33 Medium Supplemented with Bile. Trans. R. Soc. Trop. Med. Hyg..

[26] Indrayanto G., Putra G.S., Suhud F. (2021). Validation of in-vitro bioassay methods: Application in herbal drug research. Profiles Drug Subst. Excip. Relat. Methodol..

[27] Evans-Osses I., Mojoli A., Monguió-Tortajada M., Marcilla A., Aran V., Amorim M., Inal J., Borràs F.E., Ramirez M.I. (2017). Microvesicles released from *Giardia intestinalis* disturb host-pathogen response in vitro. Eur. J. Cell Biol..

[28] Lü Z., Li X., Li K., Wang C., Du T., Huang W., Ji M., Li C., Xu F., Xu P. (2021). Structure-Activity Study of Nitazoxanide Derivatives as Novel STAT3 Pathway Inhibitors. ACS Med. Chem. Lett..

[29] Indrayanto G., Putra G.S., Suhud F. (2019). Aminoguanidines: New leads for treatment of Giardia duodenalis infection. Int. J. Parasitol. Drugs Drug Resist..

[30] Singh O., Khanam Z., Misra N., Srivastava M.K. (2011). Chamomile (*Matricaria chamomilla* L.): An overview. Pharm. Rev..

[31] Shukla G., Bhatia R., Sharma A. (2016). Prebiotic inulin supplementation modulates the immune response and restores gut morphology in *Giardia duodenalis*-infected malnourished mice. Parasitol. Res..

[32] Shaaban Y., Hassan Z., Hussein R., Hassan A., Salama D. (2021). Evaluation of the role of combined prebiotic and probiotic supplements as prophylactic and therapeutic agents against experimental giardiasis. Parasitol. United J..

[33] Azagra-Boronat I., Rodríguez-Lagunas M.J., Castell M., Pérez-Cano F. (2019). Prebiotics for Gastrointestinal Infections and Acute Diarrhea. Dietary Interventions in Gastrointestinal Diseases.

[34] Gibson G.R., McCartney A.L., Rastall R.A. (2005). Prebiotics and resistance to gastrointestinal infections. Br. J. Nutr..

[35] Shoaf-Sweeney K.D., Hutkins R.W. (2008). Chapter 2 Adherence, Anti-Adherence, and Oligosaccharides: Preventing Pathogens from Sticking to the Host. Adv. Food Nutr. Res..

[36] Sousa M.C., Gonçalves C.A., Bairos V.A., Poiares-Da-Silva J. (2001). Adherence of *Giardia lamblia* trophozoites to Int-407 human intestinal cells. Clin. Diagn. Lab. Immunol..

[37] Jantscher-Krenn E., Lauwaet T., Bliss L.A., Reed S.L., Gillin F.D., Bode L. (2012). Human milk oligosaccharides reduce *Entamoeba histolytica* attachment and cytotoxicity in vitro. Br. J. Nutr..

[38] Rhoades J., Manderson K., Wells A., Hotchkiss A.T., Gibson G.R., Formentin K., Beer M., Rastall R.A. (2008). Oligosaccharide-mediated inhibition of the adhesion of pathogenic *Escherichia coli* strains to human gut epithelial cells in vitro. J. Food Prot..

[39] Guggenbichler J.P., De Bettignies-Dutz A., Meissner P., Schellmoser S., Jurenitsch J. (1997). Acidic oligosaccharides from natural sources block adherence of Escherichia coli on uroepithelial cells. Pharm. Pharm. Lett..

[40] Hotchkiss A., Buddington R. (2011). Intestinal Infections and Prebiotics: The Role of Oligosaccharides in Promoting Health. Funct. Food Rev..

[41] Ebersbach T., Andersen J.B., Bergström A., Hutkins R.W., Licht T.R. (2012). Xylo-oligosaccharides inhibit pathogen adhesion to enterocytes in vitro. Res. Microbiol..

[42] Licht T.R., Ebersbach T., Frøkiær H. (2012). Prebiotics for prevention of gut infections. Trends Food Sci. Technol..

